# The elusive perspective of a food thief

**DOI:** 10.7554/eLife.74048

**Published:** 2021-10-22

**Authors:** Claudia Zeiträg, Ivo Jacobs

**Affiliations:** Department of Cognitive Science, Lund University Lund Sweden

**Keywords:** Eurasian jay, corvids, theory of mind, desires, perspective, replication, Other

## Abstract

Eurasian jays fail to take into account the point of view and desire of other jays when hiding food they can eat later.

**Related research article** Amodio P, Farrar BG, Krupenye C, Ostojić L, Clayton NS. 2021. Little evidence that Eurasian jays protect their caches by responding to cues about a conspecific's desire and visual perspective. *eLife* **10**:e69647. doi: 10.7554/eLife.69647

How often do you think about what somebody else might know and want? The process of attributing mental states to others is called ‘theory of mind’, and is crucial for humans to successfully navigate social interactions (FeldmanHall and Shenhav, 2019). This ability not only allows us to predict one specific mental state (such as hunger), but also to integrate several so we can form a more complete picture of a person’s perspectives, desires, and knowledge. For example, it allows us to anticipate which snack someone might choose based on their cravings and what foods they can see in front of them.

For decades, researchers have been trying to find out whether non-human animals possess social cognitive skills resembling theory of mind (Premack and Woodruff, 1978; for a recent review: Krupenye and Call, 2019). While many studies have focused on whether animals attribute a specific mental state to members of the same species, very little is known about their ability to integrate multiple mental states. Now, in eLife, Nicola Clayton and colleagues from institutions in the United Kingdom, the United States, Croatia and Italy – with Piero Amodio as first author – report experiments investigating whether Eurasian jays are able to comprehend more than one mental state at a time (Amodio et al., 2021b).

Eurasian jays belong to the corvid family, which also contains ravens, crows, and magpies. Corvids have relatively big brains and remarkable intelligence, which may have evolved independently despite matching the cognitive levels of primates (Osvath et al., 2014). Like many other corvids, Eurasian jays conceal and cover food items to retrieve them later. However, this caching behaviour is sometimes observed by other jays who might decide to swoop in and steal the hidden food. The resulting dynamics between caching and pilfering resemble an arms race that appears to require complex social skills. Indeed, previous research suggests that jays can interpret other jays’ visual perspectives and desires. For example, jays preferred to cache out of view when observed by another bird (visual perspective; Legg and Clayton, 2014), and males fed their mating partners mostly food types they had not eaten recently (desire; Ostojić et al., 2013).

Amodio et al. designed two experiments to test whether a jay deciding what food to cache where can integrate the visual perspective and desire of an onlooking jay who might steal the food. In both experiments, a ‘cacher’ and an ‘observer’ were placed in adjacent aviaries. The observer was fed either macadamia nuts or peanuts in view of the cacher. This causes the observer to lose interest in the type of nut it has just eaten, and instead desire the other type of nut.

In Experiment 1, the cacher was given either the same nut the observer had just eaten, or a different type of nut that is more desired by the observer (Figure 1A). The cacher could then hide the nuts in one of two locations: a tray that the observer could see, or one that was being concealed by an opaque barrier. Amodio et al. hypothesized that if the cacher could integrate the desire and visual perspective of the observer, it would prefer to cache out of view when the nut it received was different (and therefore more desired) to the one the observer had already consumed.

**Figure 1.**
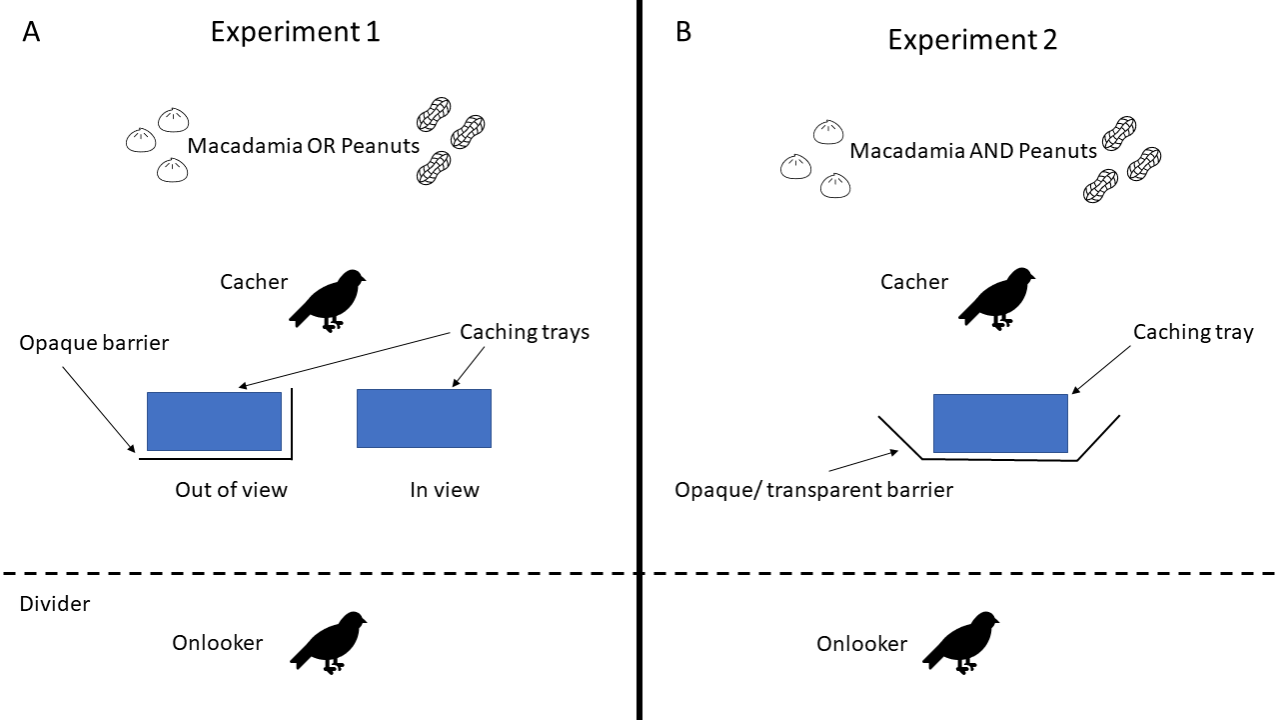
Experimental setup to test whether Eurasian jays can integrate several mental states. In both experiments, an onlooker is first pre-fed one food type in view of the cacher. (**A**) In Experiment 1, the cacher has access to one food type per trial and can cache in or out of view of the onlooker. Amodio et al. then recorded where the cacher hid the food type that had been fed to the onlooker, and the food the onlooker had not yet received (and therefore desired more). (**B**) In Experiment 2, the cacher has access to both food types but can store it in only one location that is either in view (transparent barrier) or out of view (opaque barrier) of the onlooker. In this case, Amodio et al. recorded the type of food the cacher chose to hide behind the opaque and transparent barrier.

In Experiment 2, the cacher was given both types of nuts at the same time, but they were offered a single tray to store them. In some trials, the tray was behind an opaque barrier, where the observer could not see the nuts, while in others it was behind a transparent barrier (Figure 1B). If the jays can integrate the mental state of onlookers, they should prefer to cache the nut type already fed to the observer when the barrier is transparent, and the more desired nut when the barrier is opaque.

Surprisingly, the jays had no clear preferences in either experiment, which challenges previous findings. Therefore, Amodio et al. followed up with three experiments that followed the methodology used in earlier studies that investigated sensitivity to visual perspective and desire independently (Legg and Clayton, 2014; Ostojić et al., 2017). Again, the jays showed no clear preference for caching locations or food type. Thus, Amodio et al. failed to obtain evidence that jays can integrate the visual perspective and desire of onlookers, and also found that the birds were unable to respond to just one of these mental states, contrary to past results. The experiments were performed in the same lab and with the same individuals as the preceding studies, so how is it possible that the outcomes are different?

One possible explanation is that ageing and learning effects altered the birds’ behaviour. Indeed, some jays kept the food and cached it later in their home aviaries, indicating that they may have been less motivated to cache in the ‘risky’ experimental setting when a ‘safe’ caching environment could soon become available. However, it remains unclear how future-oriented jays are when caching their food (Amodio et al., 2021a).

Other explanations for these discrepant findings reflect common issues in comparative psychology (Stevens, 2017). The tendency to publish mostly positive findings, while leaving negative result unpublished, poses a serious threat to objectivity and discourages high-risk experiments. The field also struggles with replicability due to small sample sizes, inconsistent methodologies, and ambiguous definitions (Farrar and Ostojić, 2019). The work of Amodio et al. serves as a good example for future research, which should cover multiple lines of evidence to critically examine the rigor of experiments comparing the cognitive abilities of different species.
